# Chloride of Methyl: Its Uses in Neuralgias

**Published:** 1887-07

**Authors:** 


Chloride of Methyl: Its Use in Neuralgias.—Dr. Dudley Tait (*Kansas City Medical Index*) gives the following conclusions on the use of chloride of methyl in the treatment of neuralgic affections:

1. Chloride of methyl spray may be used with great advantage in all neuralgic affections; its sole indication is *pain.*

2. It can be successfully resorted to in view of eradicating *pain* in the course of various pulmonary affections, in acute and chronic rheumatism, and also in affections similar to the writer's cramp, etc.

3. In trifacial neuralgia, precaution is of absolute necessity, but does not constitute a counter-indication.

4. One application often suffices; however, two, and exceptionally several, applications are necessary.

5. The majority of neuralgic affections are definitely cured by this treatment; success has sometimes been obtained in neuralgias symptomatic of Potts' disease, pelvic tumors, etc.

6. The slight and transient pigmentation that sometimes follows this treatment is of no importance; it disappears in the course of a few weeks.

7. Experiments seem to indicate that the analgesic properties of chloride of methyl are due to its action on the superficial terminal expansions of the nerves of the skin.

